# Lung function in severe pediatric asthma: a longitudinal study in children and adolescents in Brazil

**DOI:** 10.1186/s13601-017-0183-6

**Published:** 2017-12-15

**Authors:** Mônica Versiani Nunes Pinheiro de Queiroz, Cristina Gonçalves Alvim, Álvaro A. Cruz, Laura Maria de Lima Belizário Facury Lasmar

**Affiliations:** 1 Department of Pediatrics, School of Medicine, Federal University of Ouro Preto, Ouro Preto, Brazil; 2 Department of Pediatrics, School of Medicine, Federal University of Minas Gerais, Belo Horizonte, Brazil; 3 ProAR - Federal University of Bahia, Salvador, Brazil; 4 Departamento de Clínicas Pediátrica e do Adulto, Escola de Medicina, Universidade Federal de Ouro Preto, Rua Dois 697, Ouro Preto, MG 35400-000 Brazil

**Keywords:** Asthma in childhood and adolescence, Spirometry, Forced expiratory flow, Longitudinal study, Adherence to treatment

## Abstract

**Background:**

In severe asthma, high doses of inhaled corticosteroids (ICS) are used in order to achieve clinical and functional control. This study aimed to evaluate lung function in outpatients (children and adolescents) with severe asthma in Brazil, all of whom were treated with high doses of ICS. We evaluated all spirometry tests together and by ICS dose: 800 and > 800 µg/day.

**Methods:**

This was a 3-year longitudinal study in which we analyzed 384 spirometry tests in 65 severe asthma patients (6–18 years of age), divided into two groups by the dose of ICS (budesonide or equivalent): 800 and > 800 µg/day.

**Results:**

At baseline, the forced expiratory volume in one second (FEV_1_) and the FEV_1_/forced vital capacity (FVC) ratio were both < 80% of the predicted values in 50.8% of the patients. The median age of the patients was 10.4 years (interquartile range 7.8–13.6 years). In the sample as a whole, there were significant increases in FEV_1_% and in the FEV_1_/FVC% ratio (*p* = 0.01 and *p* < 0.001, respectively) over the course of the study. In the > 800 µg/day group, there were no statistical increases or decreases in FEV_1_, the FEV_1_/FVC ratio, or forced expiratory flow between 25 and 75% of the FVC (FEF_25–75%_), when calculated as percentages of the predicted values. However, the z-score for FEF_25–75%_ showed a statistically significant reduction, in the sample as a whole and in the > 800 µg/day group. Also in the > 800 µg/day group, there was a significant reduction in the post-bronchodilator FEV_1_% (*p* = 0.004).

**Conclusions:**

The fact that the spirometric parameters (as percentages of the predicted values) remained constant in the > 800 µg/day group, whereas there was a gain in lung function in the sample as a whole, suggests an early plateau phase in the > 800 µg/day group. However, there was some loss of lung function in the > 800 µg/day group, as evidenced by a decrease in the z-score for FEF_25–75%_, suggesting irreversible small airway impairment, and by a reduction in the post-bronchodilator FEV_1_%, suggesting reduced reversibility of airway obstruction. Among children and adolescents with severe asthma, the use of ICS doses higher than those recommended for age does not appear to improve lung function.

## Background

In most children with asthma, the disease is controlled with low to moderate doses of inhaled corticosteroids (ICS). However, approximately 5% of such children present with severe asthma that is uncontrolled or obtain asthma control only with the use of high doses of ICS, in combination with a long-acting β_2_ agonist (LABA) or leukotriene receptor antagonist, and still require frequent or prolonged treatment with oral corticosteroids [1–3]. In that population of patients, there is an increased risk of adverse reactions to the medications used, chronic morbidity, severe exacerbations, and death [1]. Pediatric asthma patients are also more likely to have presented with impaired lung growth and lung function during childhood, together with an early onset of asthma, as well as a more rapid decline in lung function, which can be lower than that expected, in adult life [4].

The finding of an abnormal trajectory through monitoring of the forced expiratory volume in one second (FEV_1_) can help identify pediatric patients at risk for abnormal lung function and irreversible airflow obstruction. Careful follow-up evaluation can identify the plateau phase and a subsequent decline [5].

The evolution of lung function in asthma patients, as determined by monitoring FEV_1_, forced vital capacity (FVC), the FEV_1_/FVC ratio, and forced expiratory flow between 25 and 75% of the vital capacity (FEF_25–75%_), has been described in longitudinal studies [5–8]. In a study conducted in the United States [5], children with mild to moderate asthma on budesonide treatment were found to show an abnormal pattern of lung growth, as determined by assessing FEV_1_ as a percentage of the predicted value (FEV_1_%), which persisted into adult life. The authors also identified an early decline in lung function in 52% of the patients. Among children with moderate asthma in Europe [8], the use of 600 µg/day of budesonide resulted in a gain in lung function, as determined by measuring the post-bronchodilator parameters related to the central and intermediate airways. However, the authors also observed a loss of lung function in the distal airways. In a study in which 38.7% of the pediatric patients had severe asthma, functional alterations in the distal airways of those patients were associated with the persistence of asthma, as evidenced by a lower FEF_25–75%_ [6]. In children with uncontrolled asthma symptoms, FEF_25–75%_ can express airway obstruction better than can FEV_1_ and the FEV_1_/FVC ratio, both of which are often normal in children with asthma [7].

The scope of ICS treatment in preventing a loss of lung function in children and adolescents with asthma has also been a concern. In a study involving children with mild to moderate asthma in the United States, the use of budesonide was not found to increase the post-bronchodilator FEV_1_%. Because those patients entered treatment between 5 and 12 years of age, the authors suggested that an irreversible loss of lung function might have occurred prior to the initiation of treatment [9]. It has been demonstrated that deficits in the FEV_1_/FVC ratio, FEV_1_, and FEF_25–75%_ observed at 2 months of age persist at 22 years of age, suggesting that reduced lung function is a risk factor for early airway obstruction in adulthood [10]. In a study involving children and adolescents with severe refractory asthma treated with 1600 µg/day of budesonide in England [11], a gain in lung function, expressed as pre-bronchodilator FEV_1_ %, was observed only in the first year of follow-up. That gain was not progressive, reaching a plateau, with a mean FEV_1_ below 80% of the predicted value, that was maintained over the following 3 years [11]. In pediatric patients with poorly controlled severe asthma, increasing the dose of ICS is recommended, because it is believed that doses > 500 µg/day of fluticasone or equivalent can be beneficial [12].

There have been few longitudinal studies of lung function in pediatric patients with severe asthma. Therefore, the objective of the present prospective study was to analyze the evolution of lung function over a 3-year period in a cohort of children and adolescents with severe asthma treated using high doses of ICS, considering all of the spirometry tests together and by dose of ICS: 800 and > 800 µg/day.

## Methods

### Study design and participants

This was a prospective cohort study in which 384 spirometry tests of 65 patients with severe asthma, obtained over a period of 3 years, were referred by pediatric pulmonologists affiliated with the Wheezing Baby Program [13], which operates under the auspices of the *Centro Multidisciplinar de Asma de Difícil Controle* (CEMAD, Multidisciplinary Center for Difficult-to-Control Asthma), a university center in the city of Belo Horizonte, in southeastern Brazil. The methods of evaluation and therapeutic management of this cohort have previously been described [14]. We excluded 192 spirometry tests: 108 because the patients had experienced exacerbations in the last 3 weeks; 33 because the tests were carried out in the learning phase; and 51 because the tests were performed less than 15 days apart.

Severe asthma is defined as asthma which requires treatment with high doses of ICS, plus a second controller (with or without oral corticosteroids) to prevent it from becoming “uncontrolled”, or which remains “uncontrolled” despite this therapy [1–3]. High doses of ICS (budesonide or equivalent) are defined as > 400 µg/day for individuals between 6 and 11 years of age and as > 800 µg/day for those over 12 years of age [3]. Because all of the patients in our cohort were being treated with a minimum of 800 µg/day of ICS (budesonide or equivalent), treatment with > 800 µg/day was classified as very-high-dose treatment.

Patients were recruited into the study between September 2010 and July 2015. At enrollment, all were in step 4 or 5 of the Global Initiative for Asthma (GINA) treatment plan and were using ≥ 800 µg/day of budesonide or equivalent [3]. All of the patients were between 6 and 18 years of age (median age, 10.4 years) and had been diagnosed with severe asthma. In all cases, the diagnosis had been confirmed after the factors associated with a lack of control (differential diagnosis, comorbidities, environmental factors, treatment adherence, and inhaler technique) had been reviewed and the treatment regimen had been adjusted according to the level of control [1–3].

The patients were evaluated periodically according to a standardized protocol that follows the recommendations of an expert panel convened by the World Health Organization to discuss severe asthma, in 2009 [1].

### Evaluation of lung function

All spirometry tests were performed at the same place and time, with a spirometer (Spirobank II; Medical International Research, Rome, Italy). The tests were performed in accordance with the recommendations of the American Thoracic Society [15]. Before and after administration of a bronchodilator (400 µg albuterol by metered-dose inhaler), we measured FEV_1_, FVC, the FEV_1_/FVC ratio, and FEF_25–75%_. Increases of 200 mL or 12% were considered significant post-bronchodilator variations in FEV_1_. The bronchodilator response was evaluated according to the proportional post-bronchodilator increase in FEV_1_ in relation to the baseline value [15].

The parameters are expressed as percentages of the values predicted value for age, gender, and height [15], as well as in z-scores, according to the Global Lung Initiative reference values [16], because the latter have been deemed valid for expressing and describing the changes over time in growing individuals [7].

### Measurement of the fraction of exhaled nitric oxide

In all patients, the fraction of exhaled nitric oxide (FeNO) was measured prior to spirometry and only when the patients were free of upper airway infections. Using a portable analyzer (NIOX MINO; Aerocrine AB, Solna, Sweden), we obtained the FeNO values at an expiratory flow rate of 50 ml/s [17].

### Optimizing treatment

At each visit, the level of asthma control was evaluated on the basis of the following parameters: daytime and nighttime symptoms; the ability to perform physical activities; and the need for rescue medication [18]. We also used the Asthma Control Test (ACT), on which a score < 20 (out of a total of 25) indicates a lack of control [19]. At each visit, the treatment regimen and specific doses were adjusted according to the level of control [2, 3, 18].

All medications were provided free of charge to the patients by the pharmacies of secondary referral centers [13]. Over the course of the study, we monitored blood pressure, growth curves, body mass index, and the basal serum level of cortisol (measured annually), as well as monitoring clinical variables to identify any adverse effects of the medication prescribed. Annual evaluations were performed by ophthalmologists and by other specialists when indicated [2].

The patients were receiving one of two types of treatment: dry-powder inhalers delivering a combination of budesonide and formoterol—Symbicort (AstraZeneca, Lund, Sweden) or Alenia (Aché Laboratórios Farmacêuticos S.A., Guarulhos, Brazil); or dry-powder or metered-dose inhalers containing fluticasone, combined with salmeterol (Seretide; GlaxoSmithKline, Stevenage, England), montelukast (Montelair; Aché Laboratórios Farmacêuticos S.A.), oral prednisolone (generic), or omalizumab (Xolair; Novartis Biociências S.A., São Paulo, Brazil).

### Inhaler technique

Every patient used a dry-powder inhaler or a metered-dose inhaler with a spacer fitted to the mouthpiece. The inhalation technique was evaluated at each visit, and the interventions proposed were reviewed at subsequent visits [1, 20].

### Adherence rate

We determined the rate of adherence to the use of the ICS by calculating the proportion of doses used in relation to the expected number of doses for each time period, on the basis of the dose counters of the devices or counting the empty capsules (for the dry-powder inhalers) and the records of the dates on which the medicines were dispensed [1, 21].

### Associated factors

In accordance with the criteria of the Allergic Rhinitis and its Impact on Asthma guidelines [22], the diagnosis of rhinitis was based on an adapted six-item clinical scale for rhinitis, each item scored from 0 to 3, corresponding to the best and worst scores, respectively [23]. Patients classified as having severe rhinitis were followed by specialists, underwent diagnostic assessments and received the necessary interventions.

We performed forearm skin prick tests using allergens obtained from ALK-Abelló (Hørsholm, Denmark), and positivity for allergic sensitization was defined as a wheal 3 mm larger than that observed for the negative control. The positive and negative controls were histamine and saline solution, respectively. We tested the following allergens [24]: *Dermatophagoides pteronyssinus*, *Dermatophagoides farinae*, *Blomia tropicalis*, *Alternaria alternata*, *Aspergillus fumigatus*, cat dander, dog dander, and cockroach allergens (from *Periplaneta americana* and *Blattella germanica*). We determined total immunoglobulin E by fluorescence enzyme immunoassay (ImmunoCAP, Phadia, Uppsala, Sweden), considering reference values by age group [25].

At each visit, we reviewed the level of environmental control in the home, considering reports of exposure to mold, passive smoking, household dust, and domestic animals, and the recommended interventions were reevaluated in subsequent visits [1, 20].

Patients with symptoms suggestive of gastroesophageal reflux disease were followed by specialists and underwent diagnostic assessment as necessary [26]. Patients for whom there were reports of emotional or behavioral disorders were referred to and monitored by specialists [18].

### Statistical analysis

Descriptive analyses were performed by calculating frequencies, means, medians, and standard deviations. Because of the considerable variability observed in the individual profiles, we employed a mixed-effects linear regression model, with a random intercept and a random slope, in our evaluation of lung function over time. The inclusion of the random effects allowed us to estimate a specific intercept for each patient, and the random slope evaluated the estimated trend for individual patients to gain or lose lung function over time.

The graphics for the longitudinal profiles were then constructed, considering all of the spirometry tests together and by treatment group: 800 µg/day of ICS; and > 800 µg/day of ICS. To smooth the longitudinal profiles and determine the mean behavior among the groups, we adopted the locally weighted scatter-plot smoothing method.

Separate models were adjusted for the pre- and post-bronchodilator values of each of the response variables, calculated as percentages of the predicted values and as z-scores: FVC, FEV_1_, FEF_25–75%_, and FEV_1_/FVC ratio. The post-bronchodilator variation in FEV_1_ was expressed in mL and in percentage. For each of these variables, we constructed two models. An initial model included only the length of follow-up (in months) and allowed us to infer the longitudinal trend (slope) for the sample as a whole. A second model was constructed in order to determine the influence that an ICS dose > 800 µg/day has on the mean behavior of lung function over time. To identify factors associated with pulmonary function, we also created a third model, which included the following covariates: gender; age at first spirometry; duration of illness; self-reported exposure to passive smoking; ACT score; occurrence of exacerbation since the previous consultation; FeNO; and length of follow-up. The models were initially adjusted for all of the covariates listed above. The covariates were selected manually: at each step, the least significant covariant (that with the highest *p* value) was removed, and the process was repeated until all non-significant covariates had been excluded. Covariates for which the estimated *p* value was less than 0.05 were considered significant. The suitability of the model was determined by visual inspection of residual plots, which did not indicate major deviations from the distributional assumptions. Data were analyzed with the program R (R Development Core Team—www.r-project.org). The level of significance was set at *p* < 0.05.

## Results

Table 1 presents the general characteristics of the patients, at enrollment and over the course of the study. At enrollment, the mean age of the patients was 10.4 years (interquartile range, 7.8–13.6 years). All of the patients were referred from pediatric pulmonology clinics, after a median follow-up of 6.1 years, having been in GINA treatment step 4 or 5 at enrollment [3]. In the 12 months prior to enrollment in the study, 94.0% of the patients had experienced severe exacerbations, 20.0% had been admitted to an intensive care unit, and 12.3% had been under continuous treatment with oral corticosteroids, all of which indicate the severity of their asthma at enrollment. At the end of the follow-up period, only one patient was classified as obese.

**Table 1:** Characteristics of children and adolescents with severe asthma (*N* = 65), at enrollment in the study and over the course of the follow-up period

Variable	At enrollment	At the end of follow-up
Female gender, *n* (%)	41 (63.0)	41 (63.0)
Age (years)^a^	10.4 (7.8; 13.6)	13.5 (8.7; 16.1)
BMI	0.31 (− 0.64; 0.88)	− 0.01 (− 0.65; 1.05)
Z-score > 3	0	1.0 (1.67)
Time followed by a pediatric pulmonologist (years)^a^	6.1 (4.3; 9.3)	–
Age of onset symptoms (years)	0.6 (1.3; 0.3)	–
Duration of disease (years)^a^	9.8 (6.1; 12.6)	–
Duration of ICS treatment (years)^a^	7.0 (4.6; 9.7)	–
Severe exacerbations in the last 12 months, *n* (%)	61 (94)	19 (29.2)
History of ICU admission due to asthma, *n* (%)	13 (20)	0
Asthma Control Test score^a^	15.5 (12.0; 20.0)	22.0 (19.0; 24.0)
Treatment adherence, %^a^	92.0 (75; 100)	93.2 (80; 100)
Inhaler technique, *n* (%)	49 (75.4)	52 (80.0)
Lung function, *n* (%)		
FEV_1_ and FEV_1_/FVC ratio ≥ 80% of predicted	32 (49.2)	52 (80.0)
FEV_1_ and FEV_1_/FVC ratio < 80% of predicted	33 (50.8)	13 (20.0)
FEF_25–75%_ < 70% of predicted	32 (49.2)	22 (44.4)
FEF_25–75%_ < 30% of predicted	1 (1.5)	1 (1.5)
Medication(s) used		
Dose of ICS (µg/day)^a,b^	800.0 (800.0; 1600.0)	876.1 (800.0; 2400.0)
Long-acting β_2_ agonist, *n* (%)	65 (100)	65 (100.0)
Leukotriene receptor antagonist, *n* (%)	11 (16.9)	40 (61.5)
Oral corticosteroid (continuous use), *n* (%)	8 (12.3)	6 (9.2)
Omalizumab, *n* (%)	0	6 (7.8)
Comorbidities		
Allergic rhinitis, *n* (%)	62.0 (95.4)	62.0 (95.4)
Allergic rhinitis score^a^	9.5 (5.3; 12.8)	6.0 (3.0; 10.0)
Gastroesophageal reflux disease, *n* (%)	9.0 (13.9)	9.0 (13.9)
Psychosocial problems, *n* (%)	10.0 (15.4)	15.0 (23.4)
Reported passive smoking in the home, *n* (%)	26.0 (40.0)	0
Fraction of exhaled nitric oxide (ppb)	22.5 (10.0; 43.3)	13.5 (4.3; 36.5)
Serum IgE (IU/mL)^a^	821.0 (299.0; 1441.0)	–
Serum IgE of 30–1500 IU/mL, *n* (%)	10 (15.4)	–
Positive skin prick test result, *n* (%)	62 (95.4)	–
*Dermatophagoides pteronyssinus*, *n* (%)	51 (78.5)	–
*Dermatophagoides farinae*, *n* (%)	43 (66.2)	–
*Blomia tropicalis*, *n* (%)	49 (75.4)	–
*Periplaneta americana*, *n* (%)	13 (20.3)	–
Cat dander, *n* (%)	7 (10.8)	–
*Blattella germanica*, *n* (%)	12 (18.5)	–
Dog dander, *n* (%)	10 (15.4)	–
Interval between spirometry tests (months)^a^		
Cohort as a whole	–	3.03 (1.87; 3.97)
> 800 µg/day subgroup	–	2.57 (1.63; 4.20)

ICU intensive care unit

^a^Median (interquartile range)

^b^Budesonide or equivalent

The median FeNO was 22.5 ppb at enrollment and 13.5 ppb at the end of the follow-up period. The majority of the patients were allergic, *D. pteronyssinus*, *D. farinae*, and *B. tropicalis* being the most common aeroallergens to which they were sensitized.

During the study, we addressed the factors that influence asthma control, such as allergic rhinitis (the median score for which dropped from 9.5 at baseline to 6.0 at the end of the study) and exposure to secondhand smoke within the home, adopting measures for its elimination. The patients with gastroesophageal reflux disease were treated by specialists, and two of those patients underwent fundoplication. All patients were reminded of the importance of correct inhalation technique and treatment adherence, both of which showed improvement over the course of the study. During the study, the median dose of ICS increased from 800 to 876.1 µg/day and the maximum dose increased from 1600 to 2400 µg/day. At enrollment, all of the patients were using a LABA in combination with the ICS. During the study, the proportion of patients using leukotriene receptor antagonists increased from 16.9 to 61.5%, whereas there was a reduction in the proportion of patients on a regimen of continuous oral corticosteroid use. Omalizumab was started in 7.8% of the patients. During the follow-up period, there was improvement in the median ACT score (which increased from 15.5 at enrollment to 22.0 at the end of the study), a reduction in the frequency of severe exacerbations, and no intensive care unit admissions, showing that treatment optimization provided clinical improvement.

Table 2 shows the pre- and post-bronchodilator values for the 384 spirometry tests evaluated. The results were analyzed in the cohort as a whole, and, to understand the influence of dose, the subgroups of patients treated with 800 and > 800 µg/day of ICS were analyzed separately [3]. The mixed-effects linear regression model provided estimates of lung function parameters, with the intercepts and their respective 95% CIs, together with the slopes, indicating the monthly variation in response.

**Table 2:** Longitudinal evaluation of spirometry tests, including pre-and post-bronchodilator values

Variables	Cohort as a whole (65 patients; 384 spirometry tests) Intercept (95% CI)	Slope (95% CI)^a^	*p*	Subgroup of patients receiving > 800 µg/day of ICS (22 patients; 57 spirometry tests) Intercept (95% CI)	Slope (95% CI)^a^	*p*
Pre-bronchodilator						
FVC (% predicted)	90.87 (87.54; 94.20)	0.09 (−0.05: 0.22)	0.20	84.65 (76.69; 92.60)	0.15 (−0.30; 0.60)	0.50
FVC (z-score)	− 0.73 (− 1.01; − 0.45)	0.00 (− 0.01; 0.01)	0.70	− 1.24 (− 1.92; − 0.55)	− 0.01 (− 0.05; 0.03)	0.60
FEV_1_ (% predicted)	81.24 (77.35; 85.13)	0.20 (0.05; 0.34)	0.01	77.07 (67.92; 86.22)	− 0.04 (− 0.60; 0.51)	0.90
FEV_1_ (z-score)	− 1.22 (− 1.58; − 0.86)	0.01 (− 0.01; 0.02)	0.40	− 1.80 (− 2.56; − 1.03)	− 0.01 (− 0.04; 0.03)	0.80
FEV_1_/FVC ratio	81.58 (79.31; 83.85)	0.12 (0.04; 0.20)	0.00	81.97 (75.49; 88.44)	− 0.11(− 0.39; 0.17)	0.50
FEV_1_/FVC ratio (z-score)	− 0.89 (− 1.21; − 0.57)	0.01 (0.00; 0.03)	0.03	− 1.15 (− 1.97; − 0.32)	0.01 (− 0.03; 0.05)	0.50
FEF_25–75 %_ (% predicted)	71.53 (65.48; 77.58)	0.43 (0.20; 0.65)	0.00	72.75 (55.33; 90.18)	− 0.24 (− 1.25; 0.76)	0.60
FEF_25–75 %_ (z-score)	− 1.43 (− 1.74; − 1.11)	− 0.02 (− 0.02; − 0.01)	0.00	− 2.02 (− 2.60; − 1.44)	− 0.02 (− 0.04; 0.00)	0.03
Post-bronchodilator						
FVC (% predicted)	93.96 (90.96; 96.96)	− 0.01 (− 0.16; 0.13)	0.90	89.45 (81.62; 97.28)	− 0.07 (− 0.47; 0.33)	0.70
FVC (z-score)	− 0.42 (− 0.71; − 0.13)	− 0.01 (− 0.02; 0.00)	0.10	− 0.89 (− 1.59; − 0.19)	− 0.02 (− 0.04; 0.01)	0.30
FEV_1_ (% predicted)	88.09 (84.61; 91.56)	0.06 (− 0.06; 0.18)	0.30	89.58 (80.62; 98.50)	− 0.48 (− 0.93; − 0.03)	0.04
FEV_1_ (z-score)	− 0.79 (− 1.11; − 0.46)	0.00 (− 0.01; 0.01)	0.10	− 1.38 (− 2.20; − 0.55)	− 0.01 (− 0.05; 0.03)	0.70
FEV_1_/FVC ratio	93.31 (90.75; 95.87)	0.09 (− 0.04; 0.23)	0.20	92.02 (85.11; 98.94)	0.04 (− 0.21; 0.30)	0.70
FEV_1_/FVC ratio (z-score)	− 0.44 (− 0.71; − 0.15)	0.02 (0.00; 0.03)	0.02	− 0.45 (− 1.43; 0.52)	0.01 (− 0.05; 0.07)	0.70
FEF_25–75 %_ (% predicted)	90.60 (83.46; 97.75)	0.11 (− 0.24; 0.46)	0.50	87.98 (69.48; 106.47)	− 0.17 (− 1.12; 0.78)	0.70
FEF_25–75 %_ (z-score)	− 0.51 (− 0.87; − 0.16)	0.01 (− 0.01; 0.02)	0.30	− 0.72 (− 1.64; 0.20)	− 0.01 (− 0.05; 0.04)	0.80
Increase in FEV_1_ (%)	9.48 (7.12; 11.84)	− 0.18 (− 0.32; − 0.03)	0.02	12.07 (6.18; 17.97)	− 0.28 (− 0.67; 0.11)	0.20

^a^Monthly variation in response

In the cohort as a whole, statistically significant increases were observed in the pre-bronchodilator FEV_1_%, in the FEV_1_/FVC ratio, and in FEF_25–75%_ (% predicted). In the > 800 µg/day subgroup, the intercept values were lower and no gain in lung function was observed for any of the parameters evaluated. However, we observed a significant reduction in the z-score for FEF_25–75%_. That phenomenon was observed in the cohort as a whole and in the > 800 µg/day subgroup.

In the longitudinal evaluation, the post-bronchodilator lung function parameters remained constant throughout the study in the cohort as a whole, with the exception of the z-score for the FEV_1_/FVC ratio, which showed a statistically significant increase. In the > 800 µg/day subgroup, most of the parameters remained constant, with no gain or loss of function, although there was a reduction in the FEV_1_%. Over the course of the study, there was a significant reduction in the FEV_1_ response to bronchodilator administration in the cohort as a whole. In the > 800 µg/day subgroup, the post-bronchodilator variation in FEV_1_ remained unchanged over the 3 years of follow-up.

**Fig. 1:**
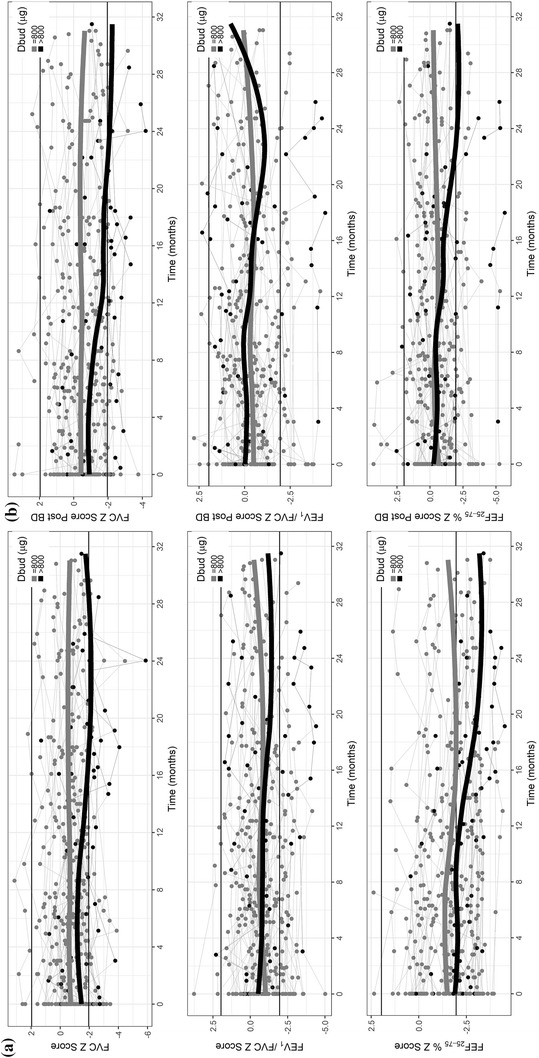
Evolution of spirometric parameters, in z-scores, in the cohort as a whole and in the subgroup receiving > 800 µg/day of ICS (budesonide or equivalent): **a** pre-bronchodilator values; **b** post-bronchodilator values. *Dbud*, budesonide-equivalent dose

Figure 1a, b depict the evolution of the pre- and post-bronchodilator spirometry parameters, expressed in z-scores, for the cohort as a whole and for the > 800 µg/day subgroup.

The evolution of the pre- and post-bronchodilator spirometry parameters, in z-scores, can be seen in Fig. 1. In the > 800 µg/day subgroup, there was a decline in lung function, especially in FEV_1_ and FEF_25–75%_, for which the values were below the lower limit of the normal range at several points.

Table 3 shows the final multivariate model of the factors associated with a decrease in FEF_25–75%_. The covariates that remained in the final model were the length of the follow-up period (*p* < 0.001), age (*p* < 0.001), and the FeNO (*p* = 0.001).

**Table 3:** Variables associated with a decline in the z-score for FEF_25–75 %_

FEF_25–75%_ (z-score)	Slope (95% CI)^a^	*p*
(Intercept)	1.74 (0.97; 2.50)	0.000
Study follow-up (months)	− 0.02 (− 0.02; − 0.01)	0.000
Age at first spirometry	− 0.26 (− 0.33; − 0.20)	0.000
FeNO	− 0.01 (− 0.01; 0.00)	0.001

^a^Monthly variation in response

## Discussion

In individuals without lung disease, a normal pattern of growth and decline in lung function, based on the FEV_1_%, has been shown to be characterized by a phase of elevation during childhood and adolescence, a plateau in young adulthood, and a subsequent decline after 30 years of age [27]. A pattern of reduced growth or early decline in lung function was demonstrated by the pre-bronchodilator FEV_1_% in patients with mild to moderate asthma in a cohort studied in the United States [5]. The authors found that lower lung function in childhood was one of the predictors of abnormal evolution and an early decline in adult life.

During follow-up at our referral outpatient clinic, the patients in our cohort showed a statistically significant increase in lung function, as determined by measurements of FEV_1_, the FEV_1_/FVC ratio, and FEF_25–75%_ (% predicted). However, in the > 800 µg/day subgroup, FEV_1_, the FEV_1_/FVC ratio, and FEF_25–75%_ remained constant over time, a behavior that suggests an early plateau. At the end of the study, the median age of our patients was 13.5 years, when they would be expected to be in the FEV_1_ gain phase.

In a four-year longitudinal study involving 47 children and adolescents (mean age, 11.2 years) with severe asthma that was refractory to treatment with 1600 µg/day of budesonide or equivalent, an early plateau phase was also observed [11]. The authors reported an annual gain in FEV_1_ of 2.6% only in the first year, with a plateau in the following 3 years.

In the Tucson birth cohort [10], which comprised children with below-normal lung function, that pattern was maintained until the age of 22. In a long-term cohort study conducted in Australia, the authors found that adults who subsequently developed chronic obstructive pulmonary disease had not presented the expected increase in lung function during adolescence [28].

The interpretation of lung function in the transition from childhood to adolescence is complex and is influenced by the multitude of predictive equations of normal values and the differences among the various age groups. Unlike the majority of biological indices in medicine, such as plasma concentrations of chemical analytes or hormones, lung function varies with age, height, gender, and ethnicity. Few equations take into account the changing relationship between lung function and height during the adolescent growth spurt [7]. The percentage of predicted does not necessarily correspond to the z-score, and, in growing individuals, the Global Lung Initiative reference equations have been deemed adequate to express and describe the changes over time [7, 16]. The use of these two reference criteria in our study (% predicted and z-score) provided an expanded view of the evolution of lung function. Although some results were discordant, the main parameter used in the assessment of obstructive diseases, the FEV_1_/FVC ratio, showed the same tendency in percentage of predicted and in z-score—an increase in the cohort as a whole and stability in the > 800 µg/day subgroup. In our study, the z-scores revealed a statistically significant reduction in the pre-bronchodilator FEF_25–75%_, in the cohort as a whole and in the > 800 µg/day subgroup.

A low FEF_25–75%_ is a sensitive indicator of small airways disease and has been shown to be useful in distinguishing patients with persistent symptoms from those with transient symptoms [7]. Small airway obstruction, evaluated by determining FEF_25–75%_, could function as a predictor of persistent asthma and of difficult-to-control asthma. Unlike FEV_1_, FEF_25–75%_ is independent of abnormalities indicative of alterations in large airways [6]. Studies have indicated that low FEF_25–75%_ in childhood is predictive of asthma in adult life, whereas it is not necessarily the case for FEV_1_ [29].

The use of FEF_25–75%_ as a diagnostic tool has limitations related to its reproducibility and interindividual variability, which are usually greater than those associated with FEV_1_ and FVC. The coefficient of variation for FEF_25–75%_ is approximately 6%, which is high but acceptable [6]. It is possible that FEF_25–75%_ is more sensitive to detecting increased airway resistance because the growth of the pulmonary parenchyma can be disproportionate or because of “distal displacement” of the middle portion of the forced expiratory curve when there is lung deflation [28]. In our study, the variables associated with a decrease in FEF_25–75%_ were the length of follow-up, age, and FeNO. These findings are consistent with those of a study involving patients with severe asthma with a median age of 10 years (range, 6–17 years), in whom changes in the obstructive pulmonary function pattern and elevated FeNO persisted over time despite high doses of ICS. This suggests that persistence of airway inflammation defines a subpopulation of pediatric patients with severe asthma [30]. The prolonged early wheezing and persistent wheezing phenotypes have been associated with the FEV_1_/FVC ratio and FEF_25–75%_ both being lower at 14–15 years of age than at 8–9 years of age, whereas the persistent wheezing phenotype has been associated with higher ratios of FeNO at 14–15 years of age [31]. Children with severe asthma have been found to show persistent, progressive airflow limitation despite treatment with high doses of ICS and other asthma controller medications. That raises major questions about corticosteroid sensitivity, as well as about whether this decline in lung function represents a reduction in the rate of lung growth or the progression of airway remodeling [32].

In a 4-year, placebo-controlled study of the use of budesonide and nedocromil sodium in children with mild persistent asthma (mean age, 9.2 years), conducted by the Childhood Asthma Management Program Research Group [9], budesonide was found to provide no gain in pulmonary function, as determined by monitoring the post-bronchodilator FEV_1_% over the course of the study, in comparison with the placebo and nedocromil sodium. The authors stated that, as a measure of lung function, post-bronchodilator FEV_1_% presents less variability over time than do pre-bronchodilator values.

The use of pre- or post-bronchodilator spirometry values to determine the degree of airway obstruction is open to discussion. Although post-bronchodilator spirometry is recommended in patients with chronic obstructive pulmonary disease, its use in asthma patients, especially in younger patients, is more controversial [6]. A high degree of reversibility of airway obstruction is a recognized marker of a lack of asthma control [4].

Among the post-bronchodilator parameters evaluated in the present study, we observed a decrease in FEV_1_% in the group of asthma patients that required even higher doses of ICS. In a study involving adults with severe asthma [33], there was a significant decline in post-bronchodilator FEV_1_ % over time (*p* < 0.001) and asthma severity was associated with a greater decline in lung function, supporting the concept that there is a specific endotype of progressive airway remodeling.

In a study involving children with moderate asthma in Europe [8], the group treated with ICS for approximately 2 years showed a significant, sustained increase in pre- and post-bronchodilator expiratory flows. However, the expiratory flows in the peripheral airways remained low, even after bronchodilator administration. The authors speculated that, even in asymptomatic individuals, residual lung function abnormalities persist, and that those abnormalities could be the result of irreversible changes or peripheral deposition of budesonide that is insufficient to reduce the distal inflammatory process.

The post-bronchodilator FEV_1_ response is a characteristic of asthma, and the magnitude of that response can decrease over the course of treatment [28]. In the present study, the z-scores for spirometry parameters remained below the lower limit of the normal range in the > 800 µg/day subgroup, even after administration of the bronchodilator. These data suggest persistent airflow limitation [34].

In one long-term study, the slopes for changes in the FEV_1_/FVC ratio indicated that a reduction in lung function occurs in early childhood [35]. Among atopic children, that reduction can occur as early as 3 years of age [10].

The patients in our study used high doses of ICS, always in combination with a LABA. Other controllers, such as leukotriene receptor antagonists and oral corticosteroids, were prescribed as needed. Omalizumab was prescribed for patients over 6 years of age who had a serum immunoglobulin E level of 30–1500 IU/mL and in whom asthma was uncontrolled despite treatment with high doses of ICS and a LABA. Over the course of the study, the mean treatment adherence rate was 93.2%. The inhalation technique, reviewed at all visits, was found to be adequate. In addition, after initial efforts to educate the patients and their relatives, exposure to secondhand smoke in the home was reportedly eliminated and the comorbidities were addressed. All of our patients were treated within the public health care system, via the CEMAD and Wheezing Baby Program, which is the first of its kind in Brazil [13]. Since 1994, it has been offering specialized treatment with medications provided free of charge. Given these considerations, we believe that the loss of lung function observed in our cohort, as determined from the FEF_25–75%_ z-score and post-bronchodilator FEV_1_%, was not due to insufficient doses of ICS, lack of other controllers, or potentially modifiable factors.

The high treatment adherence rates observed in the present study were likely due to the fact that the CEMAD is a referral center structured for the treatment of asthma patients. A study involving adult patients with severe asthma in the city of Salvador, Brazil, also showed high treatment adherence rates, which the authors suggested was attributable to the fact that those patients were under treatment at specialized centers [36].

Our study has certain limitations. The results might have limited external validity, because the patients with severe asthma were recruited from among those who had been referred to our university center and had already been followed for a median of 6 years. Our patients, all of whom were referred by pediatric pulmonologists, had not achieved control despite being in GINA treatment step 4 or 5. Nevertheless, we believe that our sample was representative of this specific patient population.

It has been postulated that an irreversible loss of pulmonary function occurs early in life [10]. Our 3-year follow-up evaluation did not include the spirometry parameters during the first years of life. At patient enrollment, the mean duration of illness was 9.8 years and the mean time of ICS use was 7.0 years.

Our results indicate that the need for higher doses of medication is associated with greater asthma severity. Over the course of our study, we observed an improvement in asthma control, as evaluated by the ACT score, although functional alterations in the distal airways persisted, as did low post-bronchodilator FEV_1_%. We observed an early plateau phase in an age group in which the majority of individuals should be gaining lung function. In the group of patients studied here, higher doses of ICS doses > 800 µg/day of budesonide or equivalent did not improve lung function parameters and the decrease in FEF_25–75%_ might reflect remodeling of the smaller airways.

## Conclusions

In conclusion, among children and adolescents with severe asthma undergoing treatment in a referral outpatient clinic in Brazil, our longitudinal evaluation evidenced a gain in lung function, based on the majority of the spirometric variables evaluated. However, there was a decline in the z-scores for pre-bronchodilator FEF_25–75%_. Nevertheless, in the > 800 µg/day subgroup, there was also a significant reduction in the post-bronchodilator FEV_1_%. The significant reduction in FEF_25–75%_ in the cohort as a whole and, more clearly, in the > 800 µg/day subgroup, suggests small airway impairment that is unresponsive to ICS and to the other controllers used. It is possible that the FEF_25–75%_ z-score is a more sensitive measure of asthma obstruction. The behavior of lung function in the > 800 µg/day subgroup (no significant gain over the 3-year follow-up period) suggests an early plateau phase. The results of our study corroborate those of previous studies showing an abnormal pattern of growth or a decline in lung function among children with asthma, provide new evidence that treatment with ICS, even at very high doses, is insufficient to prevent the problem, and underscore the fact that low FEF_25–75%_ might be a sensitive biomarker of asthma severity, potentially indicating a subpopulation of pediatric patients with severe asthma.

## Abbreviations

ICS: inhaled corticosteroids
FEV1: forced expiratory volume in one second
FVC: forced vital capacity
FEF_25–75%_: forced expiratory flow between 25 and 75% of the FVC
LABA: long-acting β2 agonist
CEMAD: Centro Multidisciplinar de Asma de Difícil Controle (Multidisciplinary Center for Difficult-to-Control Asthma)
GINA: Global Initiative for Asthma
ACT: Asthma Control Test

## References

[1] Bousquet J, Mantzouranis E, Cruz AA, Aït-Khaled N, Baena-Cagnani CE, Bleecker ER (2010). Uniform definition of asthma severity, control, and exacerbations: document presented for the World Health Organization Consultation on Severe Asthma. J Allergy Clin Immunol.

[2] Chung KF, Wenzel SE, Brozek JL, Bush A, Castro M, Sterk PJ (2014). International ERS/ATS guidelines on definition, evaluation and treatment of severe asthma. Eur Respir J.

[3] Global Strategy for Asthma Management and Prevention, Global Initiative for Asthma (GINA) 2012. http://www.ginasthma.org/.

[4] Ulrik CS (1999). Outcome of asthma: longitudinal changes in lung function. Eur Respir J.

[5] McGeachie MJ, Yates KP, Zhou X, Guo F, Sternberg AL, Van Natta ML (2016). Patterns of growth and decline in lung function in persistent childhood asthma. N Engl J Med.

[6] Siroux V, Boudier A, Dolgopoloff M, Chanoine S, Bousquet J, Gormand F, et al. Forced midexpiratory flow between 25% and 75% of forced vital capacity is associated with long-term persistence of asthma and poor asthma outcomes. J Allergy Clin Immunol. 2015. 10.1016/j.jaci.2015.10.029 26688518

[7] Piccioni P, Tassinari R, Carosso A, Carena C, Bugiani M, Bono R (2015). Lung function changes from childhood to adolescence: a seven-year follow-up study. BMC Pulm Med.

[8] Merkus PJ, van Pelt W, van Houwelingen JC, van Essen-Zandvliet LE, Duiverman EJ, Kerrebijn KF (2004). Inhaled corticosteroids and growth of airway function in asthmatic children. Eur Respir J.

[9] Szefler S, Weiss S, Tonascia J, Adkinson NF, Bender B, Cherniack R (2000). Long-term effects of budesonide or nedocromil in children with asthma. N Engl J Med.

[10] Stern DA, Morgan WJ, Wright AL, Guerra S, Martinez FD (2007). Poor airway function in early infancy and lung function by age 22 years: a non-selective longitudinal cohort study. Lancet.

[11] Sharples J, Gupta A, Fleming L, Bossley CJ, Bracken-King M, Hall P (2012). Long-term effectiveness of a staged assessment for paediatric problematic severe asthma. Eur Respir J.

[12] Bush A, Pedersen S, Hedlin G, Baraldi E, Barbato A, de Benedictis F (2011). Pharmacological treatment of severe, therapy-resistant asthma in children: what can we learn from where?. Eur Respir J.

[13] Lasmar L, Fontes MJ, Mohallen MT, Fonseca AC, Camargos P (2009). Wheezy child program: the experience of the belo horizonte pediatric asthma management program. World Allergy Organ J.

[14] de Andrade WC, Lasmar LM, Ricci CA, Camargos PA, Cruz Á (2015). Phenotypes of severe asthma among children and adolescents in Brazil: a prospective study. BMC Pulm Med.

[15] Miller MR, Hankinson J, Brusasco V, Burgos F, Casaburi R, Coates A (2005). Standardisation of spirometry. Eur Respir J.

[16] Quanjer PH, Stanojevic S, Cole TJ, Baur X, Hall GL, Culver BH (2012). Multi-ethnic reference values for spirometry for the 3-95-yr age range: the global lung function 2012 equations. Eur Respir J.

[17] Society AT, Society ER (2005). ATS/ERS recommendations for standardized procedures for the online and offline measurement of exhaled lower respiratory nitric oxide and nasal nitric oxide, 2005. Am J Respir Crit Care Med.

[18] Program NAEaP. Expert Panel Report 3 (EPR-3) (2007). Guidelines for the diagnosis and management of asthma-summary report 2007. J Allergy Clin Immunol..

[19] Roxo JP, Ponte EV, Ramos DC, Pimentel L, D’Oliveira Júnior A, Cruz AA (2010). Portuguese-language version of the Asthma Control Test. J Bras Pneumol.

[20] Bracken M, Fleming L, Hall P, Van Stiphout N, Bossley C, Biggart E (2009). The importance of nurse-led home visits in the assessment of children with problematic asthma. Arch Dis Child.

[21] Lasmar L, Camargos P, Champs NS, Fonseca MT, Fontes MJ, Ibiapina C (2009). Adherence rate to inhaled corticosteroids and their impact on asthma control. Allergy.

[22] Bousquet J, Reid J, van Weel C, Baena Cagnani C, Canonica GW, Demoly P (2008). Allergic rhinitis management pocket reference 2008. Allergy.

[23] Wilson AM, Dempsey OJ, Sims EJ, Lipworth BJ (2001). A comparison of topical budesonide and oral montelukast in seasonal allergic rhinitis and asthma. Clin Exp Allergy.

[24] Bousquet J, Heinzerling L, Bachert C, Papadopoulos NG, Bousquet PJ, Burney PG (2012). Practical guide to skin prick tests in allergy to aeroallergens. Allergy.

[25] Hamilton RG (2010). Clinical laboratory assessment of immediate-type hypersensitivity. J Allergy Clin Immunol..

[26] Gibson PG, Henry RL, Coughlan JL. Gastro-oesophageal reflux treatment for asthma in adults and children. Cochrane Database Syst Rev. 2003;(2):CD001496. 10.1002/14651858.CD001496 10.1002/14651858.CD001496 12804410

[27] Speizer FE, Tager IB (1979). Epidemiology of chronic mucus hypersecretion and obstructive airways disease. Epidemiol Rev.

[28] Tai A, Tran H, Roberts M, Clarke N, Wilson J, Robertson CF (2014). The association between childhood asthma and adult chronic obstructive pulmonary disease. Thorax.

[29] Hamid Q, Song Y, Kotsimbos TC, Minshall E, Bai TR, Hegele RG (1997). Inflammation of small airways in asthma. J Allergy Clin Immunol.

[30] Fitzpatrick AM, Gaston BM, Erzurum SC, Teague WG, National Institutes of Health/National Heart Ln, and Blood Institute Severe Asthma Research Program (2006). Features of severe asthma in school-age children: atopy and increased exhaled nitric oxide. J Allergy Clin Immunol..

[31] Duijts L, Granell R, Sterne JA, Henderson AJ (2016). Childhood wheezing phenotypes influence asthma, lung function and exhaled nitric oxide fraction in adolescence. Eur Respir J.

[32] Fitzpatrick AM, Teague WG, National Institutes of Health/National Heart, Lung, and Blood Institute’s Severe Asthma Research Program (2011). Progressive airflow limitation is a feature of children with severe asthma. J Allergy Clin Immunol.

[33] Witt CA, Sheshadri A, Carlstrom L, Tarsi J, Kozlowski J, Wilson B (2014). Longitudinal changes in airway remodeling and air trapping in severe asthma. Acad Radiol.

[34] Lødrup Carlsen KC, Hedlin G, Bush A, Wennergren G, de Benedictis FM, De Jongste JC (2011). Assessment of problematic severe asthma in children. Eur Respir J.

[35] Sears MR, Greene JM, Willan AR, Wiecek EM, Taylor DR, Flannery EM (2003). A longitudinal, population-based, cohort study of childhood asthma followed to adulthood. N Engl J Med.

[36] Souza-Machado A, Santos PM, Cruz AA (2010). Adherence to treatment in severe asthma: predicting factors in a program for asthma control in Brazil. World Allergy Organ J..

